# Xanthorrhizol: a review of its pharmacological activities and anticancer properties

**DOI:** 10.1186/s12935-015-0255-4

**Published:** 2015-10-21

**Authors:** Seok Fang Oon, Meenakshii Nallappan, Thiam Tsui Tee, Shamarina Shohaimi, Nur Kartinee Kassim, Mohd Shazrul Fazry Sa’ariwijaya, Yew Hoong Cheah

**Affiliations:** Department of Biology, Faculty of Science, Universiti Putra Malaysia-UPM, 43400 Serdang, Selangor Malaysia; Department of Chemistry, Faculty of Science, Universiti Putra Malaysia-UPM, 43400 Serdang, Selangor Malaysia; Department of Biochemistry, Faculty of Science and Technology, Universiti Kebangsaan Malaysia-UKM, 43600 Bangi, Selangor Malaysia; ZACH Biotech Depot Sdn. Bhd., 43300 Cheras, Selangor Malaysia

**Keywords:** Xanthorrhizol, *Curcuma xanthorrhiza* Roxb., Pharmacological, Anticancer

## Abstract

Xanthorrhizol (XNT) is a bisabolane-type sesquiterpenoid compound extracted from *Curcuma xanthorrhiza* Roxb. It has been well established to possess a variety of biological activities such as anticancer, antimicrobial, anti-inflammatory, antioxidant, antihyperglycemic, antihypertensive, antiplatelet, nephroprotective, hepatoprotective, estrogenic and anti-estrogenic effects. Since many synthetic drugs possess toxic side effects and are unable to support the increasing prevalence of disease, there is significant interest in developing natural product as new therapeutics. XNT is a very potent natural bioactive compound that could fulfil the current need for new drug discovery. Despite its importance, a comprehensive review of XNT’s pharmacological activities has not been published in the scientific literature to date. Here, the present review aims to summarize the available information in this area, focus on its anticancer properties and indicate the current status of the research. This helps to facilitate the understanding of XNT’s pharmacological role in drug discovery, thus suggesting areas where further research is required.

## Background

Natural products are always characterized as more drug-likely and biological friendly than totally synthetic molecules [1]. Many of them have been proven to have better compatibility with biological system and lesser side effects. New chemical entities derived from natural products have played a key role in many drug discovery programmes including anticancer, antimicrobials and anti-inflammatory drugs. They are considered as good lead compounds suitable for further modification during drug development. The World Health Organisation (WHO) estimated that annual global use of herbal medicines is about US $83 billion in 2008, indicating that natural products are important sources of new therapeutics and future medicines [2].

Studies of Lahlou addressed that alternative drug discovery methods for synthetic drugs failed to deliver many lead compounds in medicinal therapy [1]. It has been proven that many synthetic drugs have limited potential due to toxic side effects and treatment inefficiency. For instance, failure in chemotherapy is caused by dose-limiting toxicity related to drug resistance [3]. Since drug resistance is caused by human multidrug resistance associated proteins (MRPs) [4], natural product such as XNT may contribute new therapeutics that could suppress MRPs, thus improving current medication use.

To date, no comprehensive review has been done on the pharmacological activities of XNT. The present review aims to summarize the available information in this area, focus on its anticancer properties and indicate the current status of the research. This helps to facilitate the understanding of XNT’s pharmacological role in drug discovery, thus suggesting areas where further research is required.

## Discovery of xanthorrhizol (XNT)

**Fig. 1:**
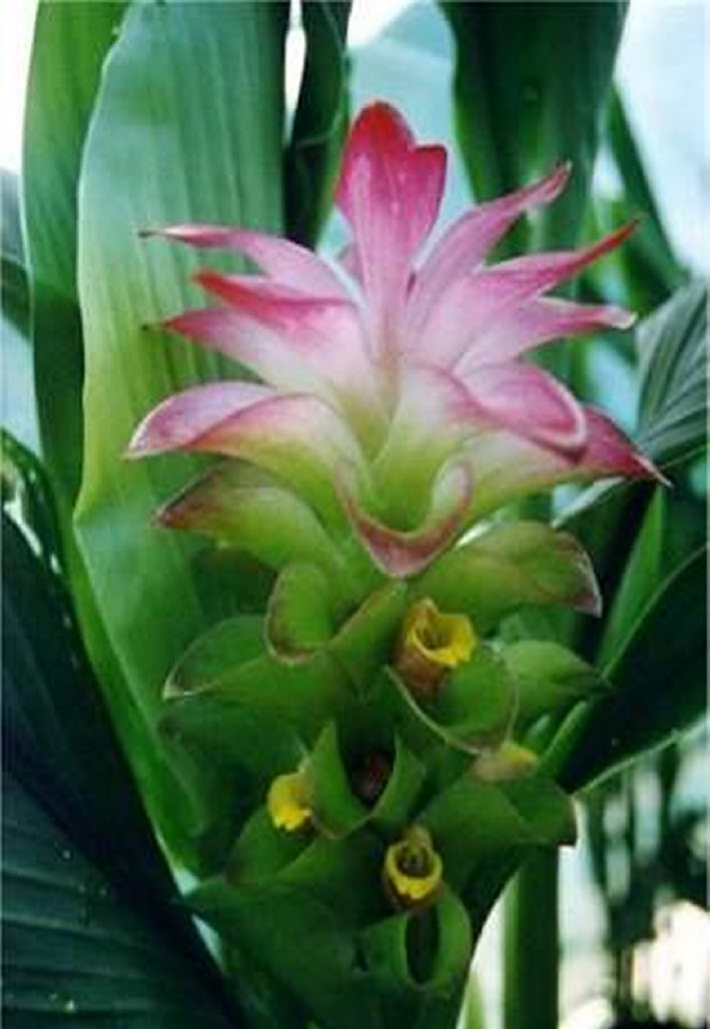
*C. xanthorrhiza* Roxb. It is a ginger-like plant of the family *Zingiberaceae.* The flowers are generally *yellow*, where the sheath is *yellow* and the aerial part is *purple*

**Fig. 2:**
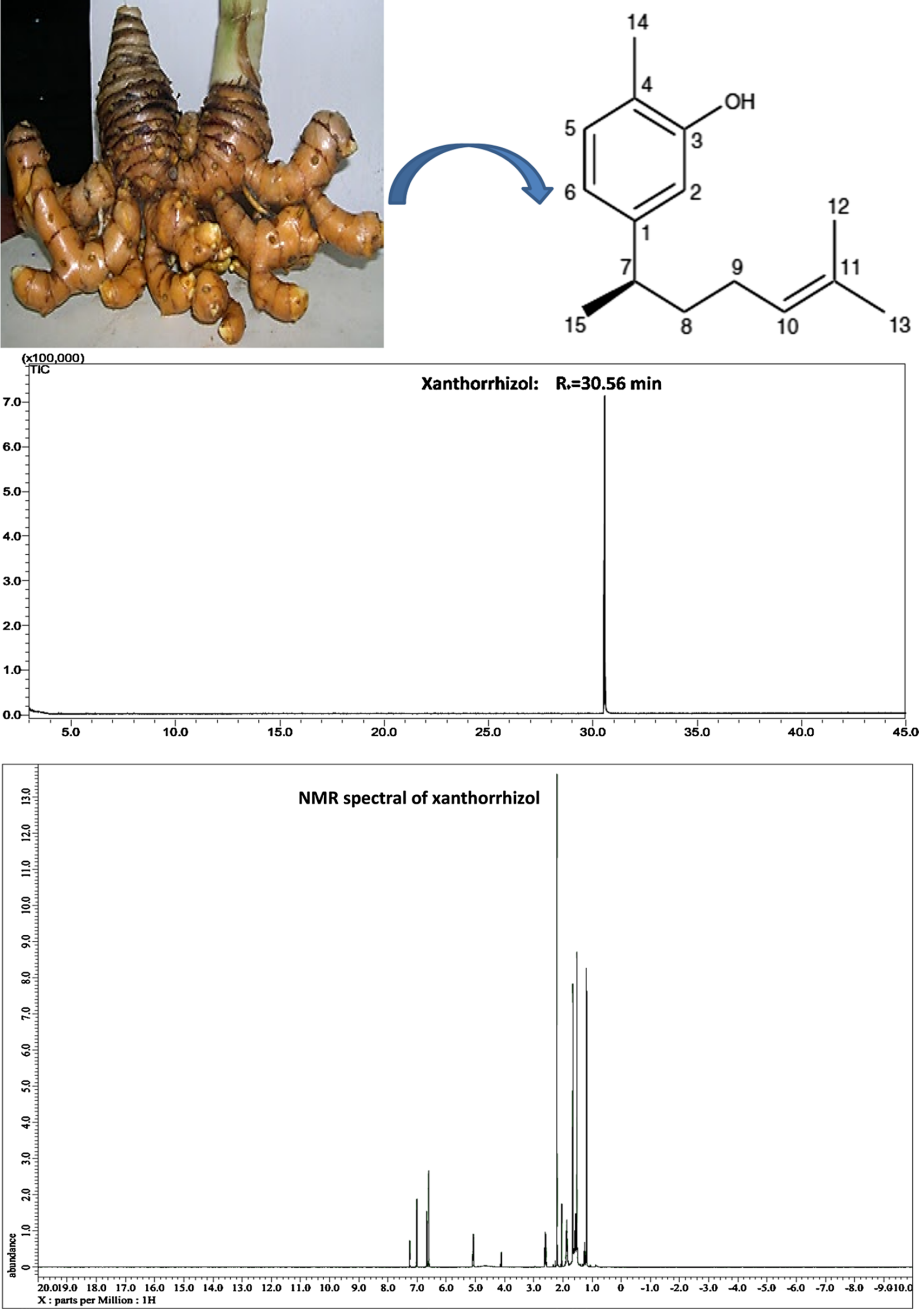
Isolation of XNT from the rhizome of *C. xanthorrhiza* Roxb. Purity of XNT is determined by GC–MS at 100 % abundance. XNT is then identified by NMR spectral, which is characterized as a bisabolane-type sesquiterpenoid compound

XNT is the most active and abundant compound isolated from the essential oil of the rhizomes of *Curcuma xanthorrhizza* Roxb. [5], also known as Java turmeric [6]. *C. xanthorrhiza* (Fig. 1) is a ginger-like plant of the family *Zingiberaceae* [7, 8], which is distributed in Southeast Asia region [8, 9]. Although it originates from Indonesia [7], it has been grown wild and cultivated in Thailand, Philippines, Sri Lanka and Malaysia [10]. It has a round tuber [7] with the dingy yellow outer skin (Fig. 2) and yellow flesh [11]. The rhizomes smell balmy and taste bitter [11].

There are several methods used to extract the essential oil and XNT including supercritical fluid carbon dioxide extraction (SCFE-CO_2_), Soxhlet extraction and percolation process [8]. According to Salea and colleagues (2014), SCFE-CO_2_ at factor combination of pressure 15 MPa, temperature 50 °C, flow rate 15 g/min and duration 60 min, has the highest XNT compared to Soxhlet and percolation extraction system. Before the introduction of SCFE-CO_2_, many researchers [6, 12–20] were still using conventional solvent extraction method to isolate XNT. Since the cost production of using SCFE-CO_2_ is much higher than conventional method, we suggest that SCFE-CO_2_ is more applicable in large-scale production in the industry.

Conventionally, the dried rhizomes of *C. xanthorrhiza* are grounded and soaked in 95 % ethanol for 2 days at room temperature [6]. The filtrate is subjected to a rotary evaporator to produce a concentrated extract under reduced pressure. Next, it is separated by silica gel column chromatography eluted with *n*-hexane–ethyl acetate solution (10:1, v/v) to give different fractions. The desired fraction is further purified by reverse phase (C18) column chromatography and eluted with 80 % methanol. The presence of XNT in each fraction is pre-identified using thin layer chromatography (TLC) [14, 18]. Then, high performance liquid chromatography (HPLC) analysis (≥98 %) [21] and gas chromatography–mass spectrometry (GC–MS) [14, 18] are used to determine the purity of XNT. Finally, succeed isolation of XNT is confirmed by NMR spectral analysis [14, 18]. It is categorized as a bisabolane-type sesquiterpenoid compound [7, 10]. The structure of XNT is shown in Fig. 2.

## Historical application of XNT

Previous studies evaluated that XNT has antimicrobial [15, 16, 19, 22–25], anti-inflammatory [10, 17, 26, 27], antioxidant [5, 17], antihyperglycemic [6], antihypertensive [21, 28], antiplatelet [29], nephroprotective and hepatoprotective [30–32], estrogenic and antiestrogenic properties [20, 33]. These pharmacological activities are summarized in Table 1.

**Table 1:** Historical application of XNT

Pharmacoactivity	Description	References
Antimicrobial	Antibacterial (*Actinomyces viscosus*, *Porphyromona gingialis*, *Streptococcus mutans*, *Staphylococcus aureus*, methicillin-resistant *S. aureus*, *Escherichia coli*, *Propionibacterium acnes*), anticandidal (*Candida albicans*, *C. glabrata*, *C. guilliermondii* and *C. parapsilosis*), antifungal (*Malassezia* species, *Aspergillus flavus*, *A. fumigatus*, *A. niger, Fusarium oxysporum, Rhizopus oryzae* and *Trichophyton mentagrophtes*)	[15, 16, 19, 22–25]
Anti-inflammatory	In vitro reduced COX-2, iNOS, TNF-α and IL-6 levels; in vivo counteracted the effect of TPA-induced ODC, COX-2 and iNOS activation in mouse skin, and prevented IkBα degradation; blocked the neurogenic and inflammatory pain response in the formalin induced pain test in rats	[10, 17, 26, 27]
Antioxidant	Suppressed H_2_O_2_-induced lipid peroxidation in rat brain homogenates, glutamate-induced neurotoxicity and ROS production; inhibited human LDL peroxidation	[5, 17]
Antihyperglycemic	Reduced the levels of insulin, glucose, FFA, TG in serum; reduced the size of epididymal fat pad and adipocyte; decreased the production of TNF-α, IL-6, IL-1β and CRP in adipose tissue, liver and muscle	[6]
Antihypertensive	Calcium antagonistic activity in rat uterus and thoracic aorta	[21, 28]
Antiplatelet	Inhibited platelet aggregation stimulated by arachidonic acid, collagen and ADP	[29]
Nephroprotective and hepatoprotective	Attenuated JNKs phosphorylation involved in MAPK signaling; inactivated NF-kB, AP-1; downregulated COX-2 and iNOS, reduced blood GPT and GOT levels	[30–32]
Estrogenic and anti-estrogenic	Upregulated pS2 and promoted EREs in MCF-7 cells; acted as partial antagonist hERα in T47D cells	[20, 33]

*COX*-*2* cyclooxygensae-2, *iNOS* inducible nitric oxide synthase, *TNF*-*α* tumor necrosis factor-alpha, *IL*-*6* interleukin-6, *TPA* 12-*O*-tetradecanoylphorbol-13-acetate, *ODC* ornithine decarboxylase, *IkBα* IkappaBalpha, *H*_*2*_ *O*_*2*_ hydrogen peroxide, *ROS* reactive oxygen species, *LDL* low-density lipoprotein, *FFA* free fatty acid, *TG* triglyceride, *IL*-*1β* interleukin-1ß, *CRP* C-reactive protein, *ADP* adenosine diphosphate, *JNK* c-Jun N-terminal kinase, *MAPK* mitogen-activated protein kinases, *NF*-*kB* nuclear factor kappaB, *AP*-*1* activator protein 1, *GPT* glutamate-pyruvate transaminase, *GOT* glutamate–oxaloacetate transaminase, *pS2* trefoil factor 1, *ERE* estrogen responsive element, *hERα* human estrogen receptor-α

### Antimicrobial properties

XNT is considered active against a variety of pathogenic microorganisms. Antimicrobial effects of XNT included antibacterial [15, 16, 22], anticandidal [19, 23] and antifungal activities [24, 25]. There have been evaluated by in vitro susceptibility tests such as minimum inhibitory concentration (MIC), minimum bactericidal concentration (MBC), minimum fungicidal concentration (MFC), NCCLS (M38-A) standard method and biofilm quantification.

Earlier study by Hwang and colleagues reported that XNT showed the highest antibacterial activity against dental caries causing bacteria (*Streptococcus* species) followed by periodontitis causing bacteria (*Actinomyces viscosus* and *Porphyromona gingialis*) [16]. XNT also strongly inhibited Gram-positive bacteria *Staphylococcus aureus,* methicillin-resistant *Staphylococcus aureus* (MRSA), Gram-negative bacteria *Escherichia coli* [34] and acne-causing bacteria *Propionibacterium acnes* [35].

Moreover, the ability of XNT in preventing dental plaque and removing oral bacterial biofilms has been demonstrated on the oral *Streptococcus mutans* biofilms in vitro [22]. Biofilms removal activities were affected by XNT concentration, exposure time and the biofilm phase growth. For example, XNT (5 µM) completely inhibited the formation of *S. mutans* biofilms at adherent growth phase, whilst XNT (50 µM) removed 76 % of biofilm at plateau accumulated phase after 60 min exposure. XNT killed *S. mutans* at planktonic growth due to its direct contact with biofilm outer layer cells [15, 22, 36]. The antimicrobial activities were induced by the capability of the hydrophobic chains of XNT to penetrate and reduce the viability of dental plaque biofilm [37].

For anticandidal activity, XNT inhibited planktonic cells of *Candida albicans* at MICs range of 1–15 µg/mL [19]. This finding is controversial with the previous result [16], where *Candida albicans* were found to be resistant to XNT. Since there is lack of information on the XNT’s condition used in the previous work [16], we infer that XNT dissolved in dimethyl sulfoxide (DMSO) [19] may enhance anticandidal activity towards *C. albicans*. The ability of XNT to prevent and kill *C. albicans* was further supported by Rukayadi and Hwang, where XNT at 8 µg/mL completely reduced *C. albicans* biofilms at adherent phase, whilst 32 µg/mL reduced 88 and 67.5 % of biofilm at intermediate and mature phase, respectively [23]. It was also active against pathogenic non-*Candida albicans* species such as *C. glabrata*, *C. guilliermondii* and *C. parapsilosis* biofilms in vitro [19, 38]. These results indicated that XNT might be used to cure biofilm-related candidal infections and treat candidiasis.

On the other hand, XNT performed antifungal activity against planktonic fungal cells such as *Malassezia* species [24] and opportunistic filamentous fungi [25]. Anti-Malassezia activity of XNT was reported in *M. furfur* and *M. pachydermatis* [24]. XNT also inhibited the conidial germination of all six filamentous fungi species such as *Aspergillus flavus*, *Aspergillus fumigatus*, *Aspergillus niger*, *Fusarium oxysporum*, *Rhizopus oryzae* and *Trichophyton mentagrophtes* based on NCCLS (M38-A) standard method. Its effect was comparable to amphotericin B [25].

Although antimicrobial mechanisms of XNT are not well understood, we believe that XNT may suppress nuclear factor kappaB (NF-kB) and mitogen-activated protein kinase (MAPK) induced by microbial infection. XNT has been demonstrated to inactivate both of them in skin cancer [26]. According to Wilken et al., infectious antigens could induce the activation of NF-kB [39]. For example, exposure of epithelial cells to *C. albicans* hyphae stimulates pro-inflammatory immune responses via NF-kB and MAPK pathways [40], which are also involved in the carcinogenesis [41, 42].

Based on epidemiologic studies, it has been estimated about 15 % of the worldwide cancer incidence is considerably related with microbial infection [43]. Chronic infection of human papilloma virus in immunocompetent hosts causes cervical carcinoma, whilst hepatitis B and C virus infection leads to hepatocellular carcinoma. Mirobes may also induce cancer incidence through opportunistic infection such as human herpes virus (HHV)-8 infection leading to Kaposi’s sarcoma [44–46]. In addition, gastric cancer secondary to *Helicobacter pylori* colonization or colon cancer may occur in certain people due to abnormal immune responses to microbes contributed by chronic inflammatory bowel disease precipitated by the intestinal microflora [44–46]. Since XNT has anticancer and antimicrobial properties, we suggest that its antimicrobial mechanism studies should be conducted not only to develop XNT as a potent antimicrobial agent, but also provides new insight on the suppression of microbes-induced cancer in the future.

### Anti-inflammatory properties

First in vitro anti-inflammatory report of XNT has been shown in lipopolysaccharide-activated mouse leukaemic monocyte macrophage cell RAW 264.7 [27]. XNT reduced cyclooxygenase-2 (COX-2) and inducible nitric oxide synthase (iNOS) activity by inhibiting the production of prostaglandin E2 (PGE2) and nitric oxide (NO) respectively in lipopolysaccharide-activated mouse macrophage cell RAW 264.7. These results indicated XNT may be a potent COX-2 and iNOS inhibitors [27], which is suggested by another anti-inflammatory assay of XNT performed in activated primary cultured microglial cells induced by lipopolysaccharide [17]. It was found to inhibit COX-2, iNOS, proinflammatory cytokine interleukin-6 (IL-6) and tumor necrosis factor-α (TNF-α) in activated microglial cells. It is clear that XNT is capable to inhibit COX-2 and iNOS as consistent with several findings [26, 27, 30], whilst IL-6 and TNF-α as consistent with recent report [6].

Further in vivo anti-inflammatory studies of XNT have been conducted in 12-*O*-tetradecanoylphorbol-13-acetate (TPA)-induced mouse acute inflammation model [26]. XNT has been reported to counteract the effect of TPA-induced ornithine decarboxylase (ODC), COX-2 and iNOS activation in mouse skin. Since pro-inflammatory proteins COX-2 and iNOS are highly associated with cutaneous inflammation, cell proliferation and skin tumor promotion [26, 47], their suppression are important to alleviate inflammation and prevent cancer [26, 41, 42, 48]. The expression of COX-2 and iNOS might be regulated by transcription factor, NF-kB, as reported in cultured cell lines and TPA-induced cutaneous inflammation in mouse skin [17, 26]. When nuclear translocation and DNA binding of NF-kB increase in response to external stimuli, NF-kB stimulates COX-2 and iNOS transcription [26, 48]. Thus, NF-kB plays a pivotal role in inflammation and tumorigenesis.

Another study postulated that XNT may exert anti-inflammatory activity by blocking the neurogenic and inflammatory pain response in the formalin induced pain test in rats [10]. It may partly contribute to the analgesic effects or antinociceptive activity. However, the detailed mechanisms have not been worked out. From the integration of findings [10, 17, 26, 27], we summarize that anti-inflammatory mechanism of XNT involved inhibition of IL-6 and TNF-α, and suppression of COX-2 and iNOS expression via NF-kB pathway resulting PGE2 and NO reduction.

### Antioxidant properties

Antioxidant properties of XNT contribute to its neuroprotective [17] and LDL oxidation inhibitory effects. XNT has been known to possess in vitro antioxidant activity against murine hippocampal neuronal HT22 cell line [17] and copper-mediated isolated human low-density lipoprotein (LDL) oxidation [5]. In murine hippocampal neuronal HT22 cell line, XNT reduced the free radical-mediated oxidative damage [17]. Its antioxidant properties exerted potent neuroprotective effects by suppressing hydrogen peroxide (H_2_O_2_)-induced lipid peroxidation in rat brain homogenates, glutamate-induced neurotoxicity and reactive oxygen species (ROS) production in HT22 cells. These results indicated that XNT could be a potent agent to treat Alzheimer’s disease and ROS associated neurological disease [17].

On the other hand, the inhibition of copper-catalysed LDL oxidation was evaluated employing thiobarbituric acid reactive substances (TBARSs) assay with human LDL as the oxidation substrate [5]. XNT strongly inhibited human LDL peroxidation in a dose-dependent manner. The presence of phenolic hydroxyl group (sesquiterpene phenol) on the bisabolene skeleton of XNT, has most probably contributed to its strong antioxidant properties by chelating Cu^2+^. This in turns may suppress the initiation of LDL oxidation and generation of free radicals at the lipoprotein [5]. We suggest that XNT might be subjected to further investigation in cardiovascular disorders because high LDL antioxidant activity could reduce the risk of heart attack. In vivo antioxidant assay could also be conducted in the future.

### Antihyperglycemic properties

In vivo antihyperglycemic effects of XNT have been demonstrated in the high-fat diet (HFD)-induced obese mice [6]. XNT and *C. xanthorrhiza* extract with standardized XNT reduced the levels of insulin, glucose, free fatty acid (FFA), and triglyceride (TG) in their serum. XNT also reduced the size of epididymal fat pad and adipocyte and decreased the production of inflammatory cytokines such as TNF-α, IL-6, interleukin-1ß (IL-1ß), and C-reactive protein (CRP) in adipose tissue, liver and muscle of HFD-induced obese mice. Thus, XNT may prevent fatty liver disease (accumulation of liver fat) and chronic inflammation [6].

These results showed that XNT’s antihyperglycemic and anti-inflammatory activities may restrict and treat type 2 diabetes, which is mainly caused by obesity-induced insulin resistance [6]. Insulin resistance is related to chronic low-grade inflammation states such as increased proinflmmatory cytokine levels. The inflammation process is initiated by the activation of TNF-α, IL-6, IL-1ß and CRP, which are known to disrupt the transduction of insulin signalling causing insulin resistance [6]. Based on this study, we reveal that XNT could suppress HFD-induced metabolic disorders including hyperglycemia, inflammation and hepatic injury by inhibiting fatty acid release from adipose tissue. We suggest that anti-obesity effects of XNT and its related mechanisms of action could be studied in the future.

### Antihypertensive properties

XNT extracted from *Iostephane heterophylla* has shown potential antihypertensive activities [28]. A preliminary study demonstrated that XNT effectively inhibited precontractions induced by calcium chloride, potassium chloride and noradrenaline in rat thoracic aorta rings. The vasorelaxation effect of XNT indicated that it may act as a calcium antagonist by reducing calcium influx into vascular smooth muscle cells in rat aorta. In fact, its calcium antagonistic activity has been illustrated earlier in isolated rat uterine smooth muscle [21]. XNT attenuated the effect of rat uterus’ tonic contraction stimulated by calcium chloride, potassium and calcium channel agonist in a dose-dependent manner. This might be due to the ability of XNT to block the voltage operated calcium influx in myometrial cells. According to Grossman and Messerli, calcium antagonists reduce blood pressure via vasodilation and decreased peripheral resistance [49]. Since calcium antagonists have been well established as basic antihypertensive drugs [50], we believe that XNT may have blood pressure-lowering effect. However, detailed antihypertensive activities and mechanisms of XNT are yet to be elucidated.

### Antiplatelet properties

In vitro antiplatelet activity of XNT (100 µg/mL) showed a strong inhibition towards platelet aggregation stimulated by arachidonic acid (100 %), collagen (81.3 %) and adenosine diphosphate (ADP) (78.6 %) in human whole blood [29]. Although previous studies reported that the antiplatelet activity of curcumin was higher than XNT [29], the potential of XNT as an antiplatelet compound should not be neglected. We suggest that its antiplatelet mechanism requires further investigation.

### Nephroprotective and hepatoprotective properties: cisplastin-induced toxicity

Nephroprotective and hepatoprotective effects of XNT have been performed in male ICR mice treated with cisplatin [30–32]. Cisplatin is a potent chemotherapeutic drug [31, 51], but the occurrence of nephrotoxicity has become the main limitation of using cisplatin-based chemotherapy [32, 52]. XNT exhibited nephroprotective effect by attenuating the increased specific gravity of kidney induced by cisplatin [32]. Cisplatin-induced kidney injury was reported as increased kidney weight, enhanced lipid peroxidation in kidney tissues, weakened filtration and excretion process of kidney, and subsequently increased blood urea nitrogen and serum creatinine levels. Pretreatment of XNT obviously restored the kidney weight to the base level and attenuated the elevated levels of blood urea nitrogen and serum creatinine. Although DNA-binding activity of NF-kB and activator protein 1 (AP-1) did not contribute to the nephroprotective effect [32], the exact mechanism has not yet been identified.

High dose of cisplatin also induces hepatotoxicity [30, 31]. Cisplatin increased DNA-binding activity of NF-kB but suppressed DNA-binding activity of AP-1. The function of NF-kB is to stimulate COX-2 and iNOS, which are associated with inflammation and toxicity. XNT pretreatment has been shown to abrogate these effects. XNT elicited hepatoprotective effects by reducing blood glutamate-pyruvate transaminase (GPT) and glutamate–oxaloacetate transaminase (GOT) levels caused by cisplatin [30]. The mechanism involved XNT’s dose-dependent attenuation of c-Jun N-terminal kinases (JNKs) phosphorylation in MAPK signaling, especially JNK1 [31]. This action may inhibit the transcription of COX-2, iNOS and transcription factor subunits (c-fos and p50). When XNT suppressed cisplatin-induced c-Fos protein expression, it may modulate the DNA-binding activity of NF-kB and AP-1, which in turns regulate COX-2 and iNOS expression. Mitochondrial apoptosis was excluded since the expression of both cytochrome c and caspase-9 was not changed [31]. Thus, it has been concluded that XNT minimized side effects of cisplatin-induced hepatotoxicity by regulating the DNA-binding activities of transcription factors NF-kB and AP-1 [30] via blocking the phosphorylation of JNK(s) [31].

It was believed that XNT exerted better suppressing effect towards cisplatin-induced nephrotoxicity [32] and hepatotoxicity than curcumin [30, 31]. At the same dose, curcumin was less effective in attenuating the elevated levels of blood urea nitrogen and serum creatinine [32]. XNT downregulated COX-2 and iNOS gene expression, but curcumin suppressed only COX-2 gene [30]. Moreover, XNT abrogated the expression of NF-kB subunit, p50 and AP-1 subunit, c-fos, but not curcumin [31]. Combined with the findings of both nephroprotective and hepatoprotective effects, we assume that XNT could be clinically applied as a suppressant of toxicity for patients administrated with high dose cisplatin to prevent kidney and liver damage.

### Estrogenic and anti-estrogenic properties

XNT has been known to possess estrogenic activity in estrogen receptor (ER)-positive MCF-7 cells during the state of hormone starvation [20, 33]. It has been reported that XNT treatment upregulated ER target gene expression, trefoil factor 1 (pS2) and promoted the interaction of ER-estrogen response elements (EREs) in MCF-7 cells. Since XNT has been proven to possess estrogenic activity in negligible estrogen level [20], we suggest that XNT could be further explored in the treatment of estrogen deficiency-induced menopausal symptoms, cardiovascular disease and osteoporosis.

In contrast, XNT was revealed as partial estrogen antagonist in T47D breast cancer cells [12]. In molecular docking simulation, the binding interaction between XNT and human estrogen receptor-α (hERα) indicated that XNT might be able to compete with estradiol. Both XNT and estradiol showed almost similar binding free-energy. Also, a strong hydrophobic interaction found between XNT and hERα may be due to the presence of hydroxyl group (1-OH) and alkyl chains, leading to its potential as partial antagonist hERα. The postulation was confirmed by pharmacophore modeling, which identified that 1-OH and alkyl chain were two important chemical features of XNT as partial antagonist hERα to strongly inhibit T47D cells. This molecular interaction with hERα also involved aromatic ring of XNT [12].

Based on the estrogenic [20, 33] and anti-estrogenic activities [12] reported, we suggest that XNT may act as a potent phytoestrogen with beneficial therapeutic potential. According to Tham et al., the partial estrogenic/anti-estrogenic behaviour is a common characteristic of phytoestrogens [53]. The estrogenic activity of phytoestrogens is 100 to 1000-fold weaker than 17β-estradiol, but its concentrations may be 100-fold higher than endogenous estrogens in the body [53]. Hence, we believe that abundant XNT molecules might act as competitive inhibitors of endogenous 17β-estradiol. XNT may block the actions of estradiol from binding to ERs of breast cancer cells, thus inhibiting tumor growth. Seeing that tumorigenesis of ER-positive luminal A cell lines (MCF-7 and T47D) can be suppressed by anti-estrogen therapy [54], XNT could be developed as a potential anti-estrogen agent.

To further study the effects of XNT as phytoestrogens in vitro, estrogen should not be excluded in experimental condition because circulating estradiol exists at all stages of the life cycle [53]. XNT may exert both estrogenic and anti-estrogenic effects on human metabolism, depending on XNT and endogenous estrogens concentration, gender and menopausal status.

## Current status of XNT

In this review, other aspects of XNT have been considered including herb-drug interaction, toxicity studies and clinical studies. XNT showed synergistic antifungal effects with amphotericin B and ketoconazole in vitro [55]. To date, only an in vivo toxicity study of XNT has been reported [56], whilst clinical study of XNT is not available so far. The details of XNT’s status have been described in the following sections.

### Herb-drug interaction

#### XNT-amphotericin B or XNT-ketoconazole

Rukayadi et al. demonstrated that XNT-amphotericin B or XNT-ketoconazole showed in vitro synergistic anticandidal effect against *Candida albicans*, *Candida glabrata*, *Candida guilliermondii*, *Candida krusei*, *Candida parapsilosis* and *Candida tropicalis* [55]. Combined XNT with amphotericin B or ketoconazole inhibited the growth of all six *Candida* species and increased cell death by several logs within 4 h [55]. We propose that XNT could be added in the formulation of conventional antifungal agents such as amphotericin B or ketoconazole to increase drug efficacy. However, the risks of side effects still need to be investigated.

#### XNT-pentobarbital

XNT at 50 mg/kg was found to prolong the pentobarbital-induced sleeping time in mice [56]. This was due to the interaction of XNT with cytochrome p450 to inhibit the metabolism of pentobarbital. Since XNT was reported to inhibit cytochrome p450 activity [56], we suggest that further studies could be conducted to elucidate its effects on hepatic drug metabolism.

### Toxicity studies

According to Yamazaki et al., a single oral administration of 500 mg/kg XNT showed no mortality in mice [56]. Since 1 mg of *C. xanthorrhiza* ethanolic extract contained 0.1238 mg of XNT [10], we estimate that up to 619 mg/kg XNT in 5 g/kg of this extract was safe to be administrated in mice. However, efficacy and safety dosage of XNT in targeted therapeutic areas are yet to be conducted in the future. Moreover, there are still lacking of studies on genotoxicity, carcinogenicity and reproductive toxicity of XNT.

### Clinical studies

To date, XNT data is unavailable in clinical pharmacology and clinical efficacy studies [57]. Not every new candidate compound discovered is fully developed and marketed [58]. We reveal that some issues restraining the availability of clinical trials are funding limitations and difficulty getting approvals from Food and Drug Administration (FDA). Frohlich supported that one of the complexities surrounding clinical research is insufficient funding to conduct modern research [59]. Aside from funding, strict ethical and regulatory compliance enforced by our social structure [59] might prolong the new drug developmental process. The major concern of clinical research is the safety and efficacy of the candidate compound [58]. It normally takes 5–6 years for a candidate drug to be submitted in a new drug application (NDA) to the FDA. Then, it required 6–10 months to complete the review of all the safety and efficacy data [58]. To overcome the delay of widespread access to new therapies, questionable data and ethical issues must be resolved. We suggest that necessary safety data, efficacy data and overall risk/benefit analysis are important key elements in successful sponsorship of XNT for future clinical studies.

## Molecular and cellular mechanisms of XNT anticancer effects

XNT is a potential suppressor of carcinogenesis. Seeing that several monophenolic groups possess cytotoxic activities, the cytotoxic effect of XNT may be contributed by its phenol group [13]. Its anticancer mechanisms are comprehensive and diverse by modulating different levels of cellular growth and apoptosis. The apoptotic morphology is usually characterized by DNA fragmentation, cell shrinkage, elongated lamellipodia and chromatin condensation [7, 18, 60–62]. Taken together with the anticancer activities that have been reported [7, 12–14, 18, 26, 41, 60–68], we suggest that the anticancer mechanisms of XNT are closely associated to its antioxidative and anti-inflammatory activities, induction of apoptosis and cell cycle arrest.

### XNT and antioxidative activity

XNT’s potent antioxidant and free radical-scavenging properties may exert chemopreventive effects on carcinogenesis. XNT was first reported to inhibit hepatic cytochrome p450 enzyme system by Yamazaki et al. [56]. Cytochrome p450 plays an important role in the oxidation and detoxification of toxic compounds [39]. When it is exposed to toxin, oxidation occurs and subsequently produces carcinogenic metabolites forming DNA adducts, which could initiate carcinogenesis [39]. Therefore, we deduce that XNT’s potent inhibitory effect on cytochrome p450 activity might suppress the initial stages of carcinogenesis by reducing ROS production in liver cells. Menon and Sudheer also presumed that membrane lipids peroxidation mediated by free radical and oxidative damage of DNA and proteins might most likely link to cancer and neurodegenerative diseases [69]. It has been shown that XNT inhibited H_2_O_2_-induced lipid peroxidation in rat brain homogenates, glutamate-induced neurotoxicity, LDL peroxidation and ROS production [5, 17]. In addition, ODC-induced reactive oxygen intermediates (ROIs) may also involve in the tumor promotion [69]. It is supported by a previous study where superoxide dismutase (SOD) and catalase suppressed ODC induction in murine mammary tumor cells [70]. Since XNT was able to suppress ODC in mouse skin cancer [26], we suggest that it may exert antipromotional activity via radical scavenging effect.

### XNT and anti-inflammatory activity

Inflammation has long been correlated with the progression of cancer [44–46, 69]. We believe that anti-inflammatory activities of XNT may inhibit tumor promotion via the modulation of transcription factor NF-kB. Increased NF-kB activation is common in many cancers because it involves the cellular pathways leading to inflammation, angiogenesis, tumorigenesis and metastasis [39]. As NF-kB is stimulated by stressful stimuli from cytokines (TNF-α and IL-1), multiple NF-kB regulated gene products (COX-2, iNOS, IL-6) might be upregulated [39].

As mentioned in Jantan et al., XNT was capable to reduce TNF-α, IL-6, IL-1β production [5]. We infer that it can switch off NF-kB activation by cytokines, thus giving anti-inflammatory effects. This is evident by several studies [17, 26, 27, 30] that showed downregulation and reduction of COX-2 and iNOS. Indeed, both enzymes are important to mediate inflammatory processes [69]. Improper upregulation can cause certain human cancers and inflammatory disorders [69].

In mouse skin with TPA-induced acute inflammation (mouse ear edema) and 19 weeks TPA-induced tumor promotion in 7,12-dimethylbenz[a]anthracene (DMBA)-initiated mouse skin, XNT suppressed cancer and inflammatory biomarkers including ODC, COX-2 and iNOS expression through MAPKs, NF-kB and or protein kinase B (Akt) [26]. XNT delayed or inhibited tumor formation, and reversed the carcinogenic process at premalignant stages [26]. Therefore, XNT’s anticancer activities may be associated with its anti-inflammatory property by inhibiting the NF-kB activity and subsequently the induction of COX-2 and iNOS.

### XNT and apoptosis induction

#### Induction of p53

Mitochondrial pathway apoptosis may be activated by p53-dependent or p53-independent pathway [71]. Several studies have proven that XNT induces apoptosis via activation of p53-dependent mitochondrial pathway as reported in HepG2 liver cancer [7], HeLa cervical cancer [61] and MCF-7 breast cancer [60]. In HeLa cervical cancer cells, XNT upregulated p53 and Bax, but not affected anti-apoptotic protein, Bcl-2 [61]. It is assumed that p53 may transcriptionally activate the pro-apoptotic Bax gene and/or repress anti-apoptotic Bcl-2 gene [72, 73]. Bax expression stimulates apoptosis, and the Bax gene product attenuates the effect of Bcl-2 protein [61, 74]. Thus, increased p53 and Bax protein expression may restore the cervical cancer cells’ sensitivity towards apoptotic stimuli [61].

These findings are in contrast with those obtained by Cheah et al. and Handayani et al., where increased expression of p53 did not induce Bax expression, but reduced Bcl-2 level in MCF-7 breast cancer [60] and HepG2 liver cancer cells [7]. When Bcl-2 expression level is low and Bax expression level remains the same [75], homodimers of Bax will regularly be produced [7] causing an increase in Bax/Bcl-2 ratio. This increases the permeability of mitochondrial membrane and stimulates cytochrome c release from mitochondria, thus initiated apoptosis [7, 39]. We speculate that XNT induced apoptosis via p53-dependent mitochondrial pathway in certain cancer cells with different effects on Bax/Bcl-2 expression.

#### Caspase activation

To initiate caspase cascade, cytochrome c binds to apoptotic protease activating factor-1 (Apaf-1) to form an apoptosome complex, which activates caspase-9 [39]. Caspase activation causes enzymatic proteolysis of cytoplasmic proteins and DNA leading to cell death. The hallmarks of caspase-dependent apoptosis as shown by XNT in cancer cells include increased mitochondrial membrane permeability and cytosolic release of cytochrome c (MDA-MB-231 invasive breast cancer [63], HCT 116 colon cancer cells [65]), activation of caspase-3 and -9 (HepG2 [7], MDA-MB-231 [63], HCT 116 cells [65]), reduced Bcl-XL expression (HepG2 [14], HCT116 cancer cells [65]), reduced Bcl-2 expression (MCF-7 [60], HepG2 [14], Tca8113 tongue cancer cells [66]), truncation of Bid (HepG2 [14], HCT116 cancer cells [65]) and cleavage of DNA repair enzyme poly-(ADP-ribose) polymerase (PARP) (HepG2 [7], MCF-7 [60], MDA-MB-231 [63], HCT116 cancer cells [65]). Indeed, PARP is a target protein of caspase-3 [65] and important to hinder cellular depletion of ATP required for apoptosis event [63]. XNT also cleaved DNA fragmentation factor 45/inhibitor of caspase-activated DNase (DFF45/ICAD) proteins in HepG2 cells [14]. These evidences suggest that the mode of XNT-induced cell death is mediated by the activation of caspase.

#### Induction of NAG-1

Pro-apoptotic non-steroidal anti-inflammatory drug-activated gene-1 (NAG-1) is a member of the transforming growth factor-β (TGF-β) superfamily [65]. It has in vitro and in vivo pro-apoptotic and antitumor effects [76–78]. During the development of human colorectal cancer and neoplastic tumors, NAG-1 is notably inhibited [65, 79, 80]. In HCT166 colon cancer study, XNT increased the expression and promoter activity of NAG-1 [65]. NAG-1 could be stimulated in either a p53-dependent [81] or p53-independent manner [82]. Although the effect of XNT on p53-induced NAG-1 expression was not investigated, enhanced NAG-1 expression by XNT promoted apoptosis in HCT116 cells. This is in agreement with previous studies [76], where NAG-1 induced apoptosis in HCT-116 colon cancer cells. It was postulated that NAG-1 gene regulation by XNT may involve inactivation of Akt/glycogen synthase kinase-3beta (GSK3β)/mammalian target of rapamycin (mTOR) signaling [65]. Although Akt inhibition activated GSK3β and subsequently enhanced NAG-1 expression [79], the exact molecular mechanisms of Akt/GSK3β/mTOR signaling on NAG-1-induced apoptosis are yet to be explored. Since NAG-1 is highly expressed in mature intestinal epithelial cells [65], we believe that it is a reliable biomarker in colon cancer screening.

#### Regulation of MAPK pathway

Subfamily members of MAPK consist of extracellular signal-regulated kinase (ERK), JNK and p38 [41]. XNT’s modulation of MAPK pathway has been shown in oral cancer in vitro [67], lung cancer and skin cancer in vivo [41]. XNT significantly increased intracellular ROS production and enhanced p38 and JNK phosphorylation in SCC-15 oral squamous cell carcinoma (OSCC) cells [67]. It was found to induce apoptosis by bypassing caspase cascade, where it cleaved PARP in the presence of caspase inhibitor. Since excessive accumulation of ROS in the cells can stimulate MAPK pathway, it is concluded that XNT induced caspase-independent apoptosis through ROS-mediated p38 MAPK and JNK activation in SCC-15 OSCC cells. XNT also prevented DMBA-induced oral carcinogenesis in hamsters [67]. We infer that XNT not only has antioxidant effects, but may also exhibit pro-oxidant activity to induce caspase-independent apoptosis during oxidative stress under pathological conditions.

In addition to oral cancer, XNT inhibited tumor nodules in a spontaneous mouse lung metastasis model and mouse skin cancer by decreasing phosphorylated ERK (pERK), JNK and p38 expression [26, 41]. XNT suppressed the activation of ERK, JNK and p38 which were persistently enhanced during the development of mouse skin papillomagenesis [26]. In mouse lung metastasis, XNT counteracted the effect of threonine-protein kinase (Raf-1), which can trigger ERK activation [41]. It also downregulated matrix metalloproteinase-9 (MMP-9) and COX-2 involved in MAPK/ERK pathway, which is an important signal cascade to inhibit mouse lung metastasis [41]. Taken together, these data provide evidence that XNT could exert antitumor effect through modulation of MAPK signalling pathway.

#### Inhibition of Akt/NF-kB pathway

XNT inhibited Akt expression and TPA-induced NF-kB activation in mouse skin cancer [26]. Since Akt is a NF-kB upstream signal factor, Akt phosphorylation can activate IkappaB kinase (IKK) followed by NF-kB, which promotes cell survival pathway [83]. We believe that Akt inhibition by XNT could suppress NF-kB. Indeed, NF-kB is inactive when it forms a cytoplasmic complex with IkappaBalpha (IkBα) [69]. NF-kB becomes active when IkBα dissociates from NF-kB through rapid phosphorylation and subsequent proteasomes degradation [26]. This activation induces apoptosis resistance, thus promoting the proliferation and survival of cancer cells [84]. XNT attenuated these effects by hindering IkBα degradation in the cytosol and blocking NF-kB translocation into the nucleus [26]. This subsequently inhibited the nuclear accumulation and DNA binding of NF-kB [26]. Based on these findings, XNT could act as Akt and NF-kB inhibitors to promote apoptosis.

In TE-1 and TE-4 esophageal squamous cancer cell lines, XNT suppressed the growth and decreased phospho-Akt (p-Akt) expression [68]. He et al. indicated that reduced Akt or p-Akt expression may inhibit NF-kB activity and subsequently induce apoptosis [83]. This is because NF-kB is correlated with various steps in the progression of malignancy included expression of anti-apoptotic protein Bcl-2 and Bcl-XL [39]. Thus, we infer that XNT exerted antiproliferative and antitumor activities in mouse skin cancer and esophageal cancer cells via inhibition of Akt/NF-kB signalling pathway.

### XNT and cell cycle arrest

In addition to apoptosis induction, growth inhibitory pathways of XNT have been demonstrated in colon cancer, esophageal cancer and tongue cancer cells. Considering cyclin D1 proto-oncogene is ultimately important to modulate the transition of G1 to S phase in various cell types [85], XNT reduced cyclin D1 expression in HCT116 colon cancer [65], TE-1 and TE-4 esophageal squamous cancer cell lines [68].

In HCT116 colon cancer cells, XNT was found to inhibit the cell cycle in the G1 or G2/M phase by triggering cyclin-dependent kinase inhibitors (CDKIs) such as p21 and p27 [65]. Cell cycle arrest in the G0/G1 and G2/M phase and increased sub-G1 peaks were closely associated with under-expression of cell cycle regulatory proteins such as cyclin A, B1, D1, cyclin-dependent kinase 1 (CDK1), CDK2, CDK4 and proliferating cell nuclear antigen (PCNA), which is a biomarker for the cell proliferation. Also, XNT stimulated G0/G1 and S + G2/M cell cycle arrest in human Tca8113 tongue cancer cell line [66]. To understand the molecular mechanisms by which XNT regulates cell cycle, extensive studies of its downstream signalling pathways will be necessary.

## Anticancer properties

XNT was first known to possess anticancer properties when it was tested on Sarcoma 180 ascites in mice [64]. Sarcoma 180 ascites is a transplantable tumor [86]. Although the antitumor activity of XNT was found to be lower than α-curcumene [64], there is lack of mechanism studies on how XNT inhibited tumor growth of Sarcoma 180 ascites.

On the other hand, antiproliferative activities of XNT have been demonstrated in many types of human breast cancer cells. These included MDA-MB-231 [18, 63], MDA-MB-453 [63], SK-BR-3 [63], MCF-7 [60], YMB-1 [13] and T47D breast cancer cell lines [12, 63]. Among the six breast cancer cell lines, only the mechanism studies of XNT towards MDA-MB-231, MCF-7 and T47D cells have been reported. We recommend that future studies should be carried out on MDA-MB-453, SK-BR-3 and YMB-1 cells to evaluate XNT’s capacity in breast cancer treatment. Furthermore, the anticancer activities of XNT have also been reported in colon cancer, cervical cancer, liver cancer, skin cancer, lung cancer, tongue cancer, oral cancer, esophageal cancer and ovarian cancer as summarized in Table 2.

**Table 2:** Anticancer properties of XNT

Types of cancer	Description	References
Breast cancer (MDA-MB-231, MCF-7, T47D, YMB-1, MDA-MB-453 and SK-BR-3 cells)	Induced mitochondrial-mediated apoptosis against MDA-MB-231 (↑ caspase-3, -9, cytochrome c and ↓ PARP-1) and MCF-7 cell apoptosis (↑ p53, ↓ Bcl-2 and PARP-1); acted as partial estrogen antagonist against T47D cell line	[12, 13, 18, 60, 63]
Colon cancer (HCT116 cells)	Induced cell cycle arrest (G0/G1, G2/M phase and increased sub-G1 peaks); mitochondrial pathway apoptosis (↑ caspase-8, -9 and-3), tBID and ↓ Bcl-XL protein, cleavage of PARP; ↑ NAG-1 may inactivate Akt pathway and subsequently suppressed GSK3β and mTOR	[65]
Cervical cancer (HeLa cells)	Induced p53 and Bax-dependent apoptosis, but not Bcl-2 and E6	[61]
Liver cancer (HepG2 cells)	Mitochondrial pathway apoptosis(↑ p53 ↓ Bcl-2 and Bcl-XL), but not Bax; caspase activation (caspase-3 and -9, not -7) involved tBid; cleavage of PARP and DFF45/ICAD proteins	[7, 14]
Skin cancer (HM3KO cells; TPA-induced tumor promotion in DMBA-initiated mouse skin)	Induced apoptosis in HM3KO cells; decreased tumor multiplicity and tumor incidence in DMBA-initiated mouse skin, suppressed ODC, COX-2 and iNOS expression through NF-kB (blocking IkBα degradation) and or Akt, inactivated ERK, p38, JNK and Akt	[26, 62]
Lung cancer (spontaneous mouse lung metastasis model)	Downregulation of MMP and COX-2 in MAPK/ERK pathway (decreased COX-2, MMP-9 and phosphorylated ERK); attenuated expression of JNK and p38; counteracted the effect of Raf-1	[41]
Tongue cancer (Tca8113 cells)	Induced cell cycle arrest in G0/G1 and S + G2/M phase; downregulated the protein expression of Bcl-2, but not Bax	[66]
Oral cancer (SCC-15 OSCC cells; DMBA-induced oral carcinogenesis in hamsters)	Caspase-independent apoptosis through ROS-mediated p38 MAPK and JNK activation in SCC-15 OSCC cells; inhibited the tumors number in buccal pouches in hamsters treated with DMBA	[67]
Esophageal cancer (TE-1 and TE-4 cells)	Reduced p-Akt and cyclin D1 expression; increased caspase-3 expression	[68]
Sarcoma 180 ascites	Lack of mechanism studies	[64]
Ovarian cancer (CaOV-3 cells)	Cytotoxic (lack of accessible information)	[62]

*PARP*-*1* poly-(ADP-ribose) polymerase-1, *tBid* truncation of bid, *NAG*-*1* non-steroidal anti-inflammatory drug-activated gene-1, *Akt* protein kinase B, *GSK3β* glycogen synthase kinase-3beta, *mTOR* mammalian target of rapamycin, *DFF45/ICAD* DNA fragmentation factor 45/inhibitor of caspase-activated DNase, *DMBA* 7,12-dimethylbenz[a]anthracene, *ERK* extracellular signal-regulated kinase, *MMP* matrix metalloproteinase, *Raf*-1 Raf-1 proto-oncogene serine/threonine-protein kinase, *OSCC* oral squamous cell carcinoma, *p*-*Akt* phospho-protein kinase B

## XNT in cancer treatment

Although XNT has been researched extensively as an antiproliferative and antitumor agent, several issues should be considered for its safety and efficacy use. For example, different biological outcomes have been reported upon the treatment of combined XNT with other compounds or drugs in breast cancer and esophageal cancer. Also, cytoselectivity of XNT has been discussed in this section.

### Breast cancer

#### XNT-curcumin interaction

When combined XNT-curcumin was added to MDA-MB-231 cells, the treatment reflected synergistic growth inhibition as compared to XNT alone [18]. Increased apoptosis index such as alteration of membrane potential, DNA condensation, DNA fragmentation and cell shrinkage have been observed. Simultaneous treatment has proven greater cytotoxic effect than sequential treatment with XNT and curcumin or vice versa [18]. Therefore, we believe that combined XNT-curcumin could be examined further for future anticancer studies.

#### XNT-tamoxifen interaction

Recent in vitro and in vivo studies of XNT-tamoxifen interaction conducted by Noomhorm and colleagues have contributed new information on breast cancer treatment translational research. XNT was found to interact with tamoxifen increasing MCF-7 proliferative activity in vivo [33].

Tamoxifen is a common non-steroidal selective ER modulator used to treat pre-menopausal and post-menopausal women with receptor-positive, ER(+)/progesterone receptor (PR)(+) breast cancers [33]. Although this hormonal therapy is recognized as a gold standard in hindering tumor recurrence of hormone-responsive breast cancer, it always results both mild and serious unfavorable side effects. Co-treatment of XNT with tamoxifen in vitro demonstrated insignificant interaction, but the effects were remarkable in tumor-bearing mice. In vitro study of XNT-tamoxifen interaction showed that there was no remarkable significant difference between XNT + tamoxifen group and tamoxifen-alone group in terms of cell number, luciferase activity, percentage S-phage cells and LC3-II expression [33].

However, in vivo results reflected that XNT interacted with tamoxifen causing increased tumor volumes, tumor size, tumor weight and protein expression of p27 (kip1) and p38 in the MCF-7 implanted athymic nude mice model [33]. Repeated dosing of XNT may accumulate its effect in stimulating cell proliferative activity of MCF-7 cells in XNT + tamoxifen group compared to a single shot XNT treatment. Since p27(kip1) was upregulated in XNT + tamoxifen group compared to the tamoxifen-alone group, it was postulated that XNT may inactivate the functional properties of p27(kip1) resulting increased tumor growth. p27(kip1) is expelled from the nucleus rendering cell growth inhibition due to phosphorylation occurs at Ser of p27(kip 1). XNT also attenuated cytotoxic effects of tamoxifen against MCF-7 implanted nude mice, most probably via p38/MAPK signaling pathway [33].

Based on the cell type and stimuli, p38 MAPK exerts pro-apoptotic and anti-apoptotic effects [87]. A study conducted by Zhou and colleagues showed that suppression of p38 MAPK inhibited tumor growth in MCF-7 xenografts [88], whilst Bacus and colleagues reported that activation of p38 MAPK was essential for MCF-7 cells’ apoptosis [89]. It exerts the pro-apoptotic action by phosphorylating and translocating Bcl-2 family’s proteins, thus causing the mitochondrial release of cytochrome c [90]. Since p27(kip1) and p38 were over-expressed in this study, in vivo tumor-promoting effect of XNT-tamoxifen interaction may be probably due to the mutation occurred leading to protein p27(kip1) and p38 malfunction. Also, this herb-drug interaction may influence the output of p38/MAPK signaling pathway, where its upregulation promoted tumor growth in MCF-7 implanted nude mice. These findings supported that *C. xanthorrhiza* consisted mainly XNT should not be used in the long-term treatment of tamoxifen treated breast cancer patients [33].

### Esophageal cancer

In TE-1 and TE-4 esophageal squamous cancer cell lines, double combination of XNT and astaxanthine or triple combination of XNT, astaxanthine and α-tocopherol exerted synergistic apoptotic effects [68]. They reduced not only the expression of p-Akt and cyclin D1, but also exhibited a higher caspase-3 expression than XNT’s treatment alone in both esophageal cancer cells. On the other hand, previous studies indicated that combined astaxanthine and α-tocopherol did not exhibit any cooperative apoptosis, where they increased the expression of p-Akt and maintained caspase-3 levels compared to control [91]. In fact, astaxanthine or α-tocopherol treatment alone was effective against TE-1 and TE-4 esophageal cancer cells, but not their combination [91]. Thus, we postulate that the addition of XNT to astaxanthine and α-tocopherol may stimulate their antiproliferative properties giving synergistic effects.

### Cytoselective and non-cytoselective effects

Cytoselective toxicity of a bioactive compound is hardly defined. A bioactive compound may be cytotoxic in certain cells while inactive towards others. XNT has been considered as cytoselective when it was tested on HeLa cervical cancer cells as compared to non-malignant Chang’s Liver and MDBK cells [61]. EC_50_ value of XNT towards both cell lines was 4.7-fold and 2.8-fold higher than HeLa cells, respectively. Also, XNT was found to be more sensitive towards MCF-7 breast cancer cells than African green monkey kidney cells (COS-7) [20].

In another study [7], XNT was moderately cytoselective against Chang’s Liver cells but lowly cytoselective towards Vero kidney cells compared to HepG2 liver cancer cells. IC_50_ value of XNT in both normal cell lines was 2.1-fold and 1.6-fold higher than HepG2 cells, respectively. Also, XNT exhibited less than twofold lower growth inhibition and cytotoxicity in both MDBK and Vero cells as compared to MDA-MB-231 breast cancer cells [63]. Surprisingly, non-cytoselective activity of XNT has been reported in normal fibroblast cell line CCD1114sk as compared to malignant melanoma HM3KO cells [62]. These results indicated that cytoselective and non-cytoselective effects of XNT depend on cell types and biological variation.

## Perspectives

Although in vivo studies reported that 500 mg/kg of XNT was not toxic to mice [56], we propose that in vivo pharmacokinetic and pharmacodynamic studies are required to further evaluate its efficacy and safety profiles before going for clinical trials. This is because the process by which a compound is absorbed, distributed, metabolized and eliminated in vivo are always far more complicated than in vitro systems [92]. The primary goals of pharmacokinetic studies are to enhance efficacy and reduce toxicity. In complex biological systems, the relationship between drug concentration at the site of action and its pharmacological response could be determined via pharmacodynamic approaches [92]. We suggest that in vivo rodent pharmacokinetic and pharmacodynamic studies of XNT should be conducted to ensure it has appropriate pharmacokinetic and pharmacodynamic properties to be investigated in clinical pharmacology and safety studies.

Since there is no information is available about genotoxicity, carcinogenicity and reproductive toxicity of XNT, future study on these areas would contribute important knowledge to the community and thus benefit/risk ratio could be determined. In fact, understanding the benefit/risk ratio is foremost important in drug prescription [93]. It is important to evaluate the toxicity of potential bioactive compound to improve the therapy effectiveness on humans and prevent devastating effects. As genotoxic effects are hardly detectable in human health, several toxicity tests could be used to assess the safety profile of XNT. In vitro and in vivo genotoxicity testing of pharmaceuticals are referred to International Conference on Harmonisation of Technical Requirements for Registration of Pharmaceuticals for Human Use (ICH)-harmonized guidance [94]. In vitro tests incorporate gene mutation in bacteria and cytogenetic evaluation of chromosomal damage and/or a test that detects gene mutation in mammalian cells. On the contrary, in vivo tests incorporate chromosomal damage using rodent hematopoietic cells and long-term assays for carcinogenicity in two different species such as mice and rats [94].

### Conclusion

The preceding sections have provided the importance of XNT as a pharmaceutical agent in disease management including cancer, infectious disease (bacteria, candida, fungi), inflammatory disease, metabolic syndrome (hyperglycemia and hypertension) and platelet disorder. It also has antioxidant, estrogenic and anti-estrogenic, nephroprotective and hepatoprotective effects. To conclude, XNT is a very potent bioactive natural compound that could fulfil the current need for new drug discovery especially in anticancer therapeutics. However, herb-drug interaction, pharmacokinetic and pharmacodynamic studies, possible genotoxicity, carcinogenicity, reproductive toxicity and clinical studies require further investigation in order to establish XNT as a standard drug.
